# Noncommunicable disease burden among HIV patients in care: a national retrospective longitudinal analysis of HIV-treatment outcomes in Kenya, 2003-2013

**DOI:** 10.1186/s12889-019-6716-2

**Published:** 2019-04-03

**Authors:** Dunstan Achwoka, Anthony Waruru, Tai-Ho Chen, Kenneth Masamaro, Evelyn Ngugi, Maureen Kimani, Irene Mukui, Julius O. Oyugi, Regina Mutave, Thomas Achia, Abraham Katana, Lucy Ng’ang’a, Kevin M. De Cock

**Affiliations:** 1 Division of Global HIV & TB, US Centers for Disease Control and Prevention (CDC), Nairobi, Kenya; 2 National AIDS and STI Control Program (NASCOP), Ministry of Health, Nairobi, Kenya; 3 University of Nairobi, Institute of Tropical and Infectious Diseases (UNITID), Nairobi, Kenya

**Keywords:** Comorbidities, Noncommunicable diseases, HIV, Kenya, Antiretroviral therapy (ART)

## Abstract

**Background:**

Over the last decade, the Kenyan HIV treatment program has grown exponentially, with improved survival among people living with HIV (PLHIV). In the same period, noncommunicable diseases (NCDs) have become a leading contributor to disease burden. We sought to characterize the burden of four major NCDs (cardiovascular diseases, cancer, chronic respiratory diseases and diabetes mellitus) among adult PLHIV in Kenya.

**Methods:**

We conducted a nationally representative retrospective medical chart review of HIV-infected adults aged ≥15 years enrolled in HIV care in Kenya from October 1, 2003 through September 30, 2013. We estimated proportions of four NCD categories among PLHIV at enrollment into HIV care, and during subsequent HIV care visits. We compared proportions and assessed distributions of co-morbidities using the Chi-Square test. We calculated NCD incidence rates and their confidence intervals in assessing cofactors for developing NCDs.

**Results:**

We analyzed 3170 records of HIV-infected patients; 2115 (66.3%) were from women. Slightly over half (51.1%) of patient records were from PLHIVs aged above 35 years. Close to two-thirds (63.9%) of PLHIVs were on ART. Proportion of any documented NCD among PLHIV was 11.5% (95% confidence interval [CI] 9.3, 14.1), with elevated blood pressure as the most common NCD 343 (87.5%) among PLHIV with a diagnosed NCD. Despite this observation, only 17 (4.9%) patients had a corresponding documented diagnosis of hypertension in their medical record. Overall NCD incidence rates for men and women were (42.3 per 1000 person years [95% CI 35.8, 50.1] and 31.6 [95% CI 27.7, 36.1], respectively. Compared to women, the incidence rate ratio for men developing an NCD was 1.3 [95% CI 1.1, 1.7], *p* = 0.0082). No differences in NCD incidence rates were seen by marital or employment status. At one year of follow up 43.8% of PLHIV not on ART had been diagnosed with an NCD compared to 3.7% of patients on ART; at five years the proportions with a diagnosed NCD were 88.8 and 39.2% (*p* < 0.001), respectively.

**Conclusions:**

PLHIV in Kenya have a high prevalence of NCD diagnoses. In the absence of systematic, effective screening, NCD burden is likely underestimated in this population. Systematic screening and treatment for NCDs using standard guidelines should be integrated into HIV care and treatment programs in sub-Saharan Africa.

## Background

The last decade has witnessed an unprecedented growth in coverage of HIV care and treatment programs globally. Expanded criteria for initiation of highly effective antiretroviral therapy (ART) for people living with HIV (PLHIV) has been associated with increased longevity and favorable treatment outcomes [1, 2]. Over the same period, noncommunicable diseases (NCDs) and associated deaths have risen steadily. At a global scale, the World Health Organization (WHO) estimates 41 million NCD-related deaths occur on an annual basis [3]. Three quarters of these deaths are in low and middle-income countries. In the general population, four major NCDs - cardiovascular diseases (including hypertension, heart attack and stroke), cancer, chronic respiratory diseases and diabetes mellitus make the largest contribution to both morbidity and mortality [4].

Sub-Saharan Africa (SSA), which is home to over half of the estimated PLHIV worldwide, is faced with a dual disease epidemic – communicable diseases and NCDs [5–7]. While several countries in SSA continue to report rapid scale-up of their national ART programs [1, 7, 8], a concomitant rise in incidence of NCDs and NCD-related deaths has also been observed over the last decade [9]. NCDs, and particularly the four aforementioned, account for over half of all hospital admissions and deaths in Kenya [6, 10]. Increased longevity of PLHIV on ART suggests likely increases in prevalence of NCDs among PLHIV in the future [1, 7, 8, 11, 12].

The burden and impact of NCDs among PLHIV in lower and middle income countries with robust ART programs is still not clearly defined [13]. Several studies examining NCDs among PLHIV have been conducted in SSA [14–18]. Most of these have involved cross-sectional surveys of facility level data, with smaller and less-representative samples. Previous national HIV treatment outcome studies in SSA have also not addressed NCDs among PLHIV [8, 19]. Additionally, there is paucity of data on the impact of noncommunicable disease burden among PLHIV from early public health approaches in HIV programming that stratified clients in care based on declining CD4 counts [20]. PLHIV in care with low CD4 counts as per prevailing national guidelines were considered eligible for HIV treatment and had ART included in their care; accordingly these “ART cohorts” were different from the corresponding clients in “pre-ART cohorts” who had higher CD4 counts than the then established thresholds for ART initiation.

Using a nationally representative sample, we sought to estimate the burden of NCDs among PLHIV enrolled in HIV care and treatment in Kenya between 2003 and 2013.

## Methods

### Study design and population

The second Longitudinal Surveillance of Treatment in Kenya (LSTIK II) was a retrospective cohort study of HIV-infected patients aged ≥15 years in Kenya, who enrolled into HIV care between October 1, 2003, and September 30, 2013. Study participants were sampled from a nationally representative random sample of 50 facilities offering ART services that had been in operation for a minimum of 15 months, and supporting at least 50 patients aged ≥15 years on ART according to the 2013 NASCOP Annual Progress Report. Our analysis was based on the cohort of patients who were enrolled in HIV care during the study period (“pre-ART cohort”), some of whom started ART in the follow-up interval between enrollment in care and data abstraction. All patients had at least 12 months of clinical follow-up prior to chart abstraction.

During the study period, there were three time periods with different ART initiation thresholds: 1st January 2003 to 31st December 2005 when the threshold for ART initiation was CD4 count < 200 cells/mm^3^; 1st Jan 2006 to 30th June 2010 when the threshold for ART initiation increased to CD4 < 250 cells/mm^3^; and 1st July 2010 to 30th September 2013 when the threshold was further increased to CD4 < 350 cells/mm^3^ [21–23].

### Data collection methods

Medical records were abstracted during October 2015 –September 2016 using a standard tool on netbook computers (Mirus Innovations, Mississauga, Ontario, Canada). Data were securely transmitted electronically to a central database in Nairobi. Data cleaning and analyses were carried out using Stata 14.2 (Stata Corporation, Texas USA).

### Measures

We described and restricted our analysis of co-morbidities to four major NCD categories - cardiovascular diseases (including hypertension, heart attack and stroke), cancer, chronic respiratory diseases (including asthma) and diabetes mellitus. These four categories are associated with over 60% of all NCD-related deaths. NCDs were measured based on documentation of any of these diagnoses at enrollment into HIV care or during the patient follow-up period. Blood pressure readings were recorded from charts. Two or more measures taken within 12 months of systolic blood pressure ≥ 140 mmHg or diastolic blood pressure ≥ 90 mmHg were defined as elevated blood pressure. The elevated blood pressure criteria were considered to be closely aligned with a clinical diagnosis of hypertension that involves multiple elevated blood pressure readings and consistent with Eighth Joint National Committee (JNC 8, 2014) recommended threshold for pharmacologic treatment of hypertension of persons aged < 60 years [24].

We conducted our analysis based on the three periods of changing CD4 count thresholds for ART initiation described above. ART drugs that constituted first line regimens among adults changed over the guideline review periods and included stavudine (d4T), zidovudine (AZT), abacavir (ABC) and tenofovir (TDF). Regimens that included lopinavir (LPV/r) were considered second line.

### Statistical analysis

We estimated proportions of NCDs among PLHIV at enrollment into HIV care, and during subsequent follow-up visits. We compared proportions and assessed distributions of baseline demographic and clinical characteristics by sex using Wald adjusted Pearson’s Chi- Square test. We used the Cox regression-based test for equality of survival curves by ART status and tested for proportional-hazards assumption. We assessed for differences in failure rates using weighted survival curves, adjusting for age at enrollment. Data were survey-set before analyses. Data were assumed to be missing at random; we did not impute the data. The percentages with an NCD were weighted to account for sampling. All estimates were adjusted to account for sampling design and missing data. Analyses were carried out in Stata 14.2 (Stata Corporation, Texas USA).

### Ethical considerations

This study was approved by the Kenya Medical Research Institute’s Scientific and Ethics Review Unit, the Kenyatta National Hospital, University of Nairobi Ethics Review Committee as part of a nested study and by the Committee on Human Research of the University of California, San Francisco. This study was reviewed according to the Centers for Disease Control and Prevention (CDC) human research protection procedures and was determined to be and approved as research.

## Results

### Study population characteristics

**Fig. 1:**
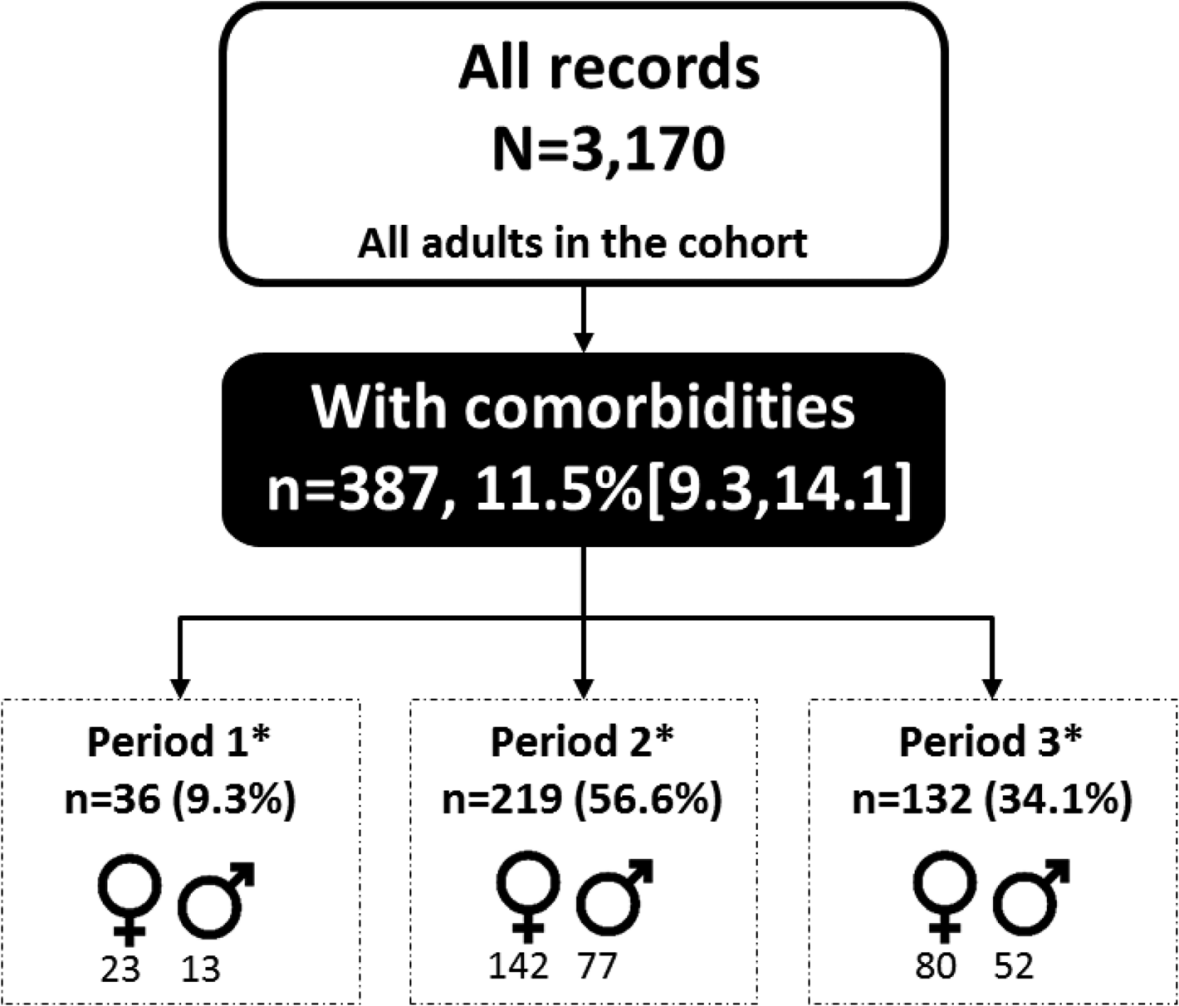
Proportion of patients by ART status, Longitudinal Surveillance of Treatment in Kenya, 2016

A total of 3170 patient records were analyzed (Fig. 1), with over two thirds of records (2115) constituting women. At the time of data abstraction, slightly over half (52.1%) of patients were aged < 35 years; women were more likely to be in this younger age group (*p* < 0.001). The majority (68.3%) of patients were employed; men were more likely to be in formal or informal employment than women (*p* < 0.001). Half (51.1%) of patients were married or cohabiting, 12% were widowed, 7.8% divorced/separated, and 13.5% single or never married. There was a significant difference in marital status between men and women (*p* < 0.001); 64.7% (95% CI: 55.9, 72.7) of men were married compared to 44.2% (95% CI: 39.9, 48.7), of women (Table 1).

**Table 1:** Distribution of characteristics of adults in care by sex, Longitudinal Surveillance of Treatment in Kenya, 2016 (*N* = 3170)

Characteristics	Total		Women		Men		*p*-value
	No.	Column (Col %) [95% CI]	No.	Col % [95% CI]	No.	Col % [95% CI]	
Total	3170	100	2115		1055		
Age (years)							< 0.001
Under 35 years	1658	52.1 [49.5,54.6]	1250	58.6 [56.0,61.2]	408	39.1 [35.4,42.9]	
35+ years	1512	47.9 [45.4,50.5]	865	41.4 [38.8,44.0]	647	60.9 [57.1,64.6]	
Employment							< 0.001
Formal and informal employment	881	68.3 [62.5,73.5]	507	60.8 [55.3,66.2]	374	81.4 [73.3,87.5]	
Unemployed	446	31.7 [26.5,37.5]	351	39.2 [33.8,44.7]	95	18.6 [12.5,26.7]	
Marital status							< 0.001
Married/cohabiting	1693	51.1 [45.8,56.4]	979	44.2 [39.9,48.7]	714	64.7 [55.9,72.7]	
Widowed	399	12 [9.5,15.0]	346	15.6 [12.2,19.7]	53	4.8 [3.4,6.8]	
Divorced/separated	254	7.8 [6.3,9.6]	192	8.9 [7.2,11.0]	62	5.5 [4.0,7.6]	
Single/Never married	416	13.5 [11.1,16.3]	313	15.6 [12.6,19.1]	103	9.3 [7.2,12.1]	
Missing marital	408	15.6 [9.2,25.3]	285	15.7 [10.1,23.4]	123	15.6 [7.5,29.6]	
Entry point							0.435
OPD/TB clinic	2077	64.4 [57.6,70.7]	1385	64.1 [57.1,70.6]	692	64.9 [57.7,71.5]	
IPD	341	11.2 [8.0,15.5]	225	10.8 [7.5,15.2]	116	11.9 [8.4,16.7]	
Others/not documented	752	24.5 [18.6,31.5]	505	25.1 [18.9,32.5]	247	23.2 [17.4,30.2]	
WHO stage							0.131
Stage I/II	127	3.8 [2.3,6.2]	85	3.9 [2.4,6.2]	42	3.7 [2.1,6.5]	
Stage III/IV	716	21.5 [18.0,25.4]	435	20.1 [16.8,24.0]	281	24.1 [19.4,29.6]	
Missing WHO	2327	74.7 [70.4,78.6]	1595	76 [71.9,79.6]	732	72.2 [66.0,77.6]	
CD4 categories							0.036
< 200	138	4.4 [3.3,6.0]	75	3.5 [2.6,4.7]	63	6.3 [4.2,9.4]	
200–250	37	1.1 [0.8,1.6]	24	1.1 [0.7,1.6]	13	1.2 [0.6,2.2]	
251–350	41	1.3 [0.9,1.8]	25	1.1 [0.7,1.8]	16	1.6 [0.8,3.0]	
351–500	79	2.8 [2.1,3.7]	49	2.6 [1.8,3.7]	30	3.1 [1.9,5.1]	
> 500	145	5 [3.9,6.3]	112	5.6 [4.3,7.2]	33	3.7 [2.5,5.7]	
Missing	2730	85.4 [82.6,87.9]	1830	86.1 [83.2,88.6]	900	84.1 [80.1,87.5]	
ART status							0.874
On ART	2170	63.9 [57.2,70.0]	1440	64 [58.5,69.2]	730	63.6 [53.9,72.3]	
Non-ART	1000	36.1 [30.0,42.8]	675	36 [30.8,41.5]	325	36.4 [27.7,46.1]	
Regimen							0.050
D4T containing regimen	758	36.5 [31.8,41.5]	506	36.9 [32.3,41.8]	252	35.7 [29.4,42.6]	
AZT containing regimen	606	26.9 [23.1,31.0]	417	28.4 [24.3,32.8]	189	23.8 [19.3,29.0]	
ABC containing regimen	7	0.5 [0.2,1.2]	4	0.5 [0.1,1.7]	3	0.5 [0.1,1.5]	
LPV/r containing regimen	8	0.4 [0.1,0.9]	8	0.6 [0.2,1.4]	0	0	
TDF containing regimen	778	35.8 [30.7,41.1]	496	33.6 [28.7,39.0]	282	40 [32.8,47.6]	
Enrolment guidelines period							0.070
01Jan2003 to 31Dec2005	243	9 [6.7,12.0]	175	9.3 [6.8,12.6]	68	8.5 [5.8,12.3]	
01Jan2006 to 30Jun2010	1681	54.3 [49.8,58.8]	1132	55.4 [50.6,60.2]	549	52.2 [47.1,57.2]	
01Jul2010 to 30Sep2013	1246	36.6 [31.3,42.3]	808	35.3 [30.1,40.9]	438	39.3 [33.2,45.8]	
Comorbidities at any time							0.308
With comorbidities	387	11.5 [9.3,14.1]	245	11.1 [8.7,14.0]	142	12.4 [9.9,15.4]	
Without comorbidities	2783	88.5 [85.9,90.7]	1870	88.9 [86.0,91.3]	913	87.6 [84.6,90.1]	

In this cohort, 63.9% of patients had initiated ART by the time of data abstraction, with no significant differences by sex (*p* = 0.874). Just over half of the patients in the cohort had been initially enrolled in care during 01 January 2006 to 30 June 2010 (54.3%); only 9% had been enrolled during the 01 Jan 2003 to 31 Dec 2005 guideline period. No differences were observed by sex across the three guideline enrollment periods (*p* = 0.070). Similar proportions of patients were on d4T and TDF containing regimens, (36.5% [95% CI: 31.8, 41.5] vs 35.8% [95% CI: 30.7, 41.1], respectively). A quarter of the patients (26.9%) had been on an AZT containing regimen. Only 0.4 and 0.5% of patients were on a LPV/r or ABC containing regimen respectively at the time of data abstraction. No significant difference was noted in regimen type between men and women (*p* = 0.05). Most patients did not have a documented WHO stage (74.7%) or CD4 count (85.4%) (Table 1).

### NCDs among PLHIV

In this cohort, 387/3170 (weighted percentage of 11.5%) (95% CI: 9.3, 14.1), had evidence of any NCD in their HIV clinical care record. No difference between the proportion of men and women with an NCD (*p* = 0.308) was observed (Table 1).

**Fig. 2:**
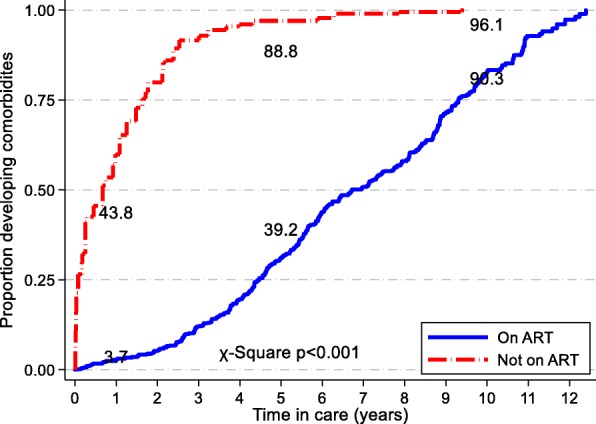
Proportion of patients developing comorbidities any time and during follow-up by ART status, Longitudinal Surveillance of Treatment in Kenya, 2016 (*N* = 3170)

The proportion of patients with a documented diagnosis of an NCD among PLHIV not on ART rose sharply in the first few years of follow-up compared to the otherwise gentle trajectory and longer duration observed for PLHIV on ART (*p* < 0.001). PLHIV who had not yet initiated ART were more likely to have an NCD diagnosis at one and five years of follow up. At one year of follow up 43.8% of PLHIV not on ART had been diagnosed with an NCD compared to 3.7% of patients on ART; at five years the proportions with a diagnosed NCD were 88.8 and 39.2% (p < 0.001), respectively (Fig. 2).

Overall NCD incidence was 35.1 per 1000 person years. Men had an overall NCD incidence of 42.3 per 1000 person years (95%CI: 35.8, 50.1) compared to 31.6 (95%CI: 27.7, 36.1) in women. The highest incidence rates were observed among 45–54 and ≥ 55 year olds at 57.5 (95%CI: 46.7, 70.9) and 55.0 (95%CI: 38.5, 78.7) per 1000 person years respectively. The 15–24 year age band had the lowest incidence rate at 21.0 per 1000 person years (95%CI: 13.8, 31.9). No significant differences in NCD incidence rates were seen based on marital or employment status, (Table 2).

**Table 2:** Incidence rates of Non Communicable Diseases (NCDs) per 1000 person years by ART status, Longitudinal Surveillance of Treatment in Kenya, 2016 (N = 3170)

Characteristics	On ART		Incidence/1000 person years [95% CI]		
	n/N	Percent [95% CI]	All	On ART	Non-ART
All	2170/3170	63.9 [57.2,70.0]	35.1[31.6, 38.9]	34.5 [31.0, 38.5]	42 [29.4–60.1]
Sex					
Female	1440/2115	64 [58.5,69.2]	31.6[27.7, 36.1]	31.1 [27.0, 35.7]	38.4 [24.8–59.5]
Male	730/1055	63.6 [53.9,72.3]	42.3[35.8, 50.1]	41.7 [35.1, 49.7]	52 [28–96.6]
Age at enrolment (years)					
15–24	261/456	53.2 [46.3,59.9]	21.0[13.8, 31.9]	19.7 [12.4, 31.2]	30.2 [11.3–80.4]
25–34	794/1202	61.3 [54.5,67.8]	26.1[21.4, 31.8]	25.0 [20.3, 30.9]	38.5 [21.3–69.6]
35–44	678/935	67.5 [59.1,74.8]	35.9[30.0, 43.0]	34.8 [28.8, 42.0]	55.4 [29.8–103]
45–54	319/428	70.1 [59.7,78.8]	57.5[46.7, 70.9]	57.0 [46.0, 70.7]	66.9 [27.9–160.8]
55 +	118/149	76.2 [67.4,83.2]	55.0[38.5, 78.7]	59.4 [41.5, 84.9]	no data
Marital status					
Ever married/cohabited	1717/2346	70.6 [66.6,74.3]	36.9[33.0, 41.3]	36.3 [32.3, 40.8]	45.7 [30.6–68.2]
Single/Never married	271/416	64 [57.4,70.1]	31.9[23.1, 44.0]	32.6 [23.4, 45.4]	23.1 [5.8–92.4]
Employment					
Formal and informal employment	666/881	72.7 [67.0,77.8]	43.8[37.5, 51.2]	43.8 [37.4, 51.3]	45.1 [21.5–94.6]
Unemployed	329/446	71.9 [64.0,78.6]	45.9[36.2, 58.2]	42.8 [33.2, 55.1]	100.2 [50.1–200.3]

**Fig. 3:**
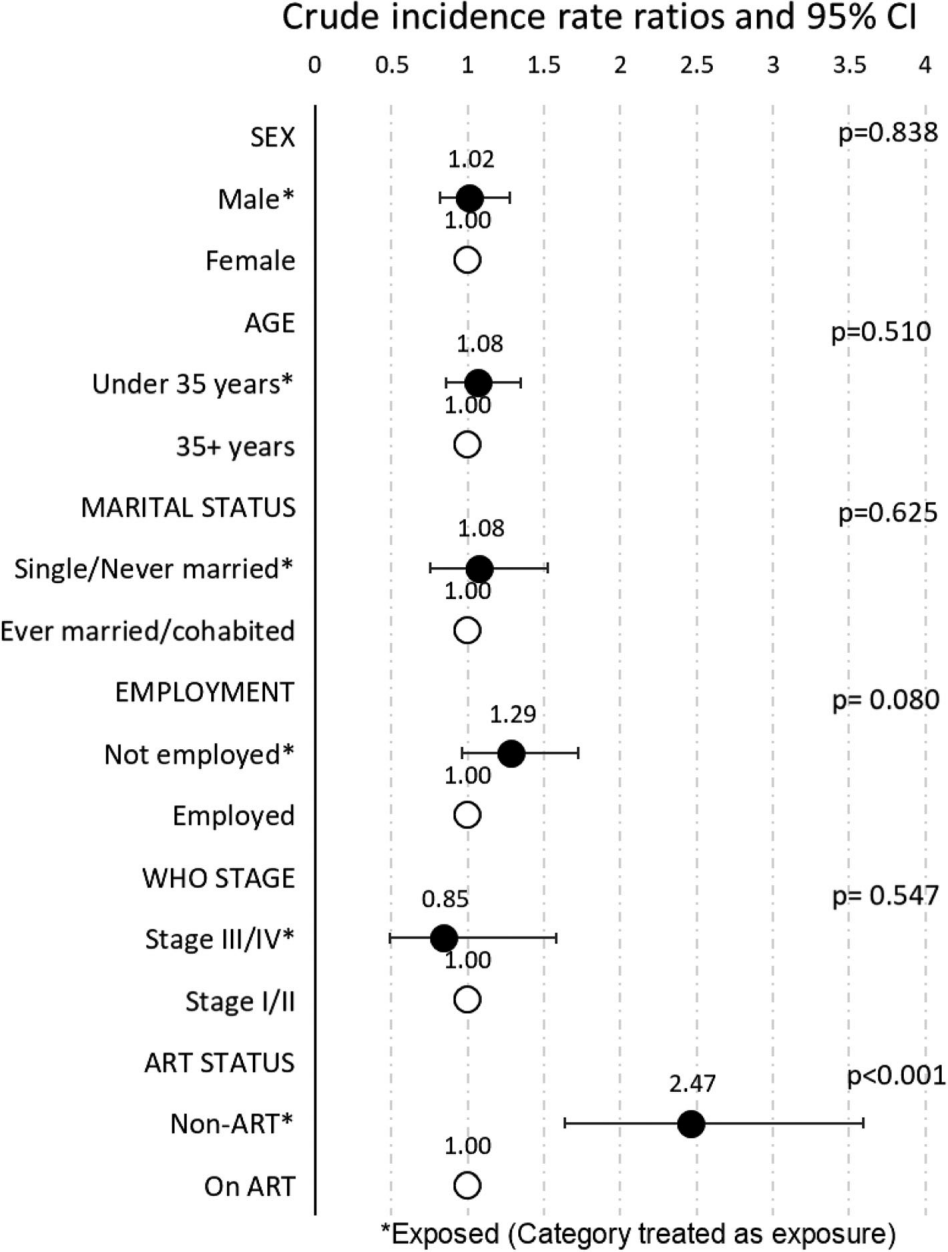
Crude incidence rate ratios for Non Communicable Diseases (NCDs) during follow-up by selected characteristics among those who have any NCD, Longitudinal Surveillance of Treatment in Kenya, 2016 (*n* = 387)

Among the 387 PLHIV with an NCD, the crude incidence rate ratio (crude IRR) for development of NCDs during follow up was 2.47 (95%CI: 1.6, 3.6) for PLHIV not initiated ART as compared with PLHIV who had initiated ART [*p* < 0.001]), (Fig. 3). Crude IRR for NCDs among men was similar to that among women (IRR = 1.02, *p* = 0.84). There was no difference in NCD crude IRR between PLHIV aged < 35 years compared to PLHIV aged ≥35 years (*p* = 0.51). No difference was detected between single/never married PLHIV and those who were married/cohabiting (IRR = 1.08, *p* = 0.62). Similarly, crude IRR of developing NCDs was no different among PLHIV who were not employed during follow up versus PLHIV who were employed (IRR = 1.29, *p* = 0.08). WHO staging comparing advanced disease staging (stage III/IV) to early disease staging (stage I/II) had a crude IRR of 0.85 (p = 0.5). Age-adjusted analysis revealed no further effect for all IRRs previously described.

### NCDs burden

#### Cardiovascular disease

We found that among PLHIV with any recorded NCD, 347/387, weighted percentage of 88.9% (95%CI 81.5, 93.5) were found to have a documented record of any cardiovascular disease (CVD) including hypertension. CVD was more frequent in persons on ART 93.9% (95%CI 90.0, 96.3) vs 53.9% (95%CI 30.7, 75.6) not on ART respectively (*p* = 0.03), (Table 3). Most identified cases of CVD were associated with elevated blood pressure.

**Table 3:** Distribution of Non Communicable Diseases (NCDs) during care by ART status, Longitudinal Surveillance of Treatment in Kenya, 2016 (n = 387)

Comorbidities	Total		On ART		non-ART		*p*-value
	No.	Col % [95% CI]	No.	Col % [95% CI]	No.	Col % [95% CI]	
Total^a^	387		346		41		
Cardiovascular Disease							
No cardiovascular disease	40	11.1 [6.5,18.5]	24	6.1 [3.7,10.0]	16	46.1 [24.4,69.3]	0.025
Cardiovascular disease	347	88.9 [81.5,93.5]	322	93.9 [90.0,96.3]	25	53.9 [30.7,75.6]	
Blood Pressure							0.030
No elevated BP^b^	44	12.5 [7.6,19.9]	27	7.2 [4.6,11.1]	17	49.4 [25.5,73.5]	
Elevated BP	343	87.5 [80.1,92.4]	319	92.8 [88.9,95.4]	24	50.6 [26.5,74.5]	
Diabetes Mellitus							0.437
No diabetes	378	97.9 [95.3,99.1]	339	98.5 [96.7,99.4]	39	93.7 [68.6,99.0]	
Diabetes	9	2.1 [0.9,4.7]	7	1.5 [0.6,3.3]	2	6.3 [1.0,31.4]	
Chronic Respiratory Disease							0.147
No asthma	378	97.7 [95.1,98.9]	340	98.7 [96.6,99.5]	38	90.7 [75.5,96.8]	
Asthma	9	2.3 [1.1,4.9]	6	1.3 [0.5,3.4]	3	9.3 [3.2,24.5]	
Cancer							0.278
No cancer	384	98.9 [95.2,99.8]	343	98.8 [94.4,99.8]	41	100	
Cancer	3	1.1 [0.2,4.8]	3	1.2 [0.2,5.6]	0	0	

^a^Includes other NCD categories not listed in the table: renal failure [4], and other, not specified [9]

^b^Elevated blood pressure: Two or more measures of systolic blood pressure ≥ 140 mmHg or diastolic blood pressure ≥ 90 mmHg taken within 12 months

##### Elevated blood pressure

Among PLHIV with any recorded NCD, 343/387, weighted percentage of 87.5% (95%CI 80.1, 92.4) were found to have two or more elevated blood pressure readings taken < 12 months apart (our proxy measure of hypertension). Among patients with an NCD comorbidity, elevated blood pressure was more frequent in persons on ART 92.8% (95%CI 88.9, 95.4) vs 50.6% (95%CI 26.5, 74.5) not on ART respectively (p = 0.03), (Table 3). Although serial elevated blood pressures were detected among 343 patients, only 17 (0.5%) had a documented diagnosis of hypertension in their medical record (results not shown).

#### Diabetes mellitus

Only 9/387, a weighted percentage of 2.1% (95%CI: 0.9, 4.7) of PLHIV with NCD had documented diabetes mellitus. Compared by ART status, no significant difference was observed between PLHIV on ART and those not on ART, (*p* = 0.44) (Table 3).

#### Chronic respiratory diseases

We found 9/387, a weighted percentage of 2.3% (95%CI 1.1, 4.9) of PLHIV with NCD had a documented diagnosis of asthma. Compared to patients on ART, there was no difference in documented asthma among non-ART patients; 1.3% (95%CI 0.5, 3.4) vs 9.3% (95%CI 3.2, 24.5) respectively, (*p* = 0.15) (Table 3).

#### Cancer

Any form of cancer was documented among 3/387, weighted percentage of 1.1% (95%CI 0.2, 4.8) of PLHIV with NCD with no statistical difference between patients on ART 1.2% (95%CI 0.2, 5.6) and those not on ART 0%, (*p* = 0.28) (Table 3).

## Discussion

This evaluation describes the burden of NCDs among HIV patients from a nationally representative survey of HIV care and treatment in Kenya prior to the national implementation of ART for all PLHIV irrespective of CD4 count. This evaluation was conducted in the context of a rising burden of NCDs in Africa and a rapid scale up of antiretroviral therapy coverage that has contributed to increased life expectancy among PLHIV [2, 14, 25–27]. In this study overall incidence rates for diagnosed NCDs were lower amongst those on ART compared to those not on ART. It is possible that this can be attributed to differences in health seeking behaviors, health care access, or socioeconomic or other factors associated with delayed initiation of ART. This finding is consistent with other studies that have shown increased prevalence of NCD risk factors among PLHIV not on antiretroviral treatment [28, 29]. Compared to other studies that suggest social economic deprivation as a predictor of NCDs risk factors among PLHIV [18, 30], our study did not detect a difference based on employment status.

The WHO’s recommendation to expand ART eligibility to all persons diagnosed with HIV was adopted in Kenya in 2016 [31]. Clinical parameters of WHO stage and baseline CD4 have previously been associated with NCD risk [32]. In our study, WHO staging and baseline CD4 showed no significant associations with NCDs risk, although documentation was incomplete.

In other countries, an increased risk of developing NCDs in PLHIV has been associated with exposure to certain ART drugs like stavudine, efavirenz and protease inhibitors [33–36]. A meta-analysis showed that exposure to ART drugs independently increases risk of metabolic and cardiovascular diseases [37]. However, our study and a study conducted in Zimbabwe conducted found no significant association between either ART drug class or duration of exposure and NCDs [14]. This discrepancy may result from limitations in detecting NCDs in our study, specific ARTs in use during this study period, or differences between populations.

The convergence of a dual burden of NCDs and communicable diseases in SSA is not in question [2, 6, 17, 27]. The burden of hypertension and cardiovascular disease regardless of HIV status remains substantial [38, 39]. Several studies have shown evidence of increased blood pressure and hypertension among PLHIVs on ART; an indication that a distinct difference exists in the characterization of cardiovascular disease between PLHIVs and non-PLHIVs [37, 40].

By using two elevated blood pressure readings within a 12 month interval (as a proxy for hypertension), our study found elevated blood pressure to be the most common (87.5%) among the 4 selected NCDs in our study population. In comparison, only 0.5% of these patients had a recorded diagnosis of hypertension. This discrepancy highlights the need for systematic screening NCDs in this population. The population of PLHIV in care that had been diagnosed with diabetes mellitus was comparable to that of the general population with raised blood glucose (2.1% vs 1%) [10]. Our study findings are similar to those of several studies and population based NCDs surveys in SSA [10, 15, 38]. There is a need to emphasize cardiovascular and metabolic risk factor assessment at all clinical visits, especially for PLHIV in older age groups [6, 13, 17, 41].

Our study found a lower prevalence of chronic respiratory diseases, including asthma, among PLHIV enrolled in care when compared to estimates for the general population derived from a separate national survey of NCDs (2.3% vs 8.5%) [10]. Although noted to be a lower prevalence, deliberate screening for findings and risk factors associated with chronic respiratory conditions such as smoking and occupational hazards should be incorporated in routine screening [42]. Of note, most facilities did not perform spirometry for respiratory disease evaluation.

Cancers are the second largest cause of NCD-related deaths and account for 7% of overall mortality in Kenya [6]. In our study, the prevalence of cancer among PLHIV enrolled in care was 1.1%. In an era of increased access to ART, systematic reviews among PLHIV indicate steadily declining rates of AIDS defining malignancies among PLHIV with most cancer diagnoses now being pre-cancerous [40]. Screening of cancers, such as cervical cancer, however remains important and cost-effective when integrated into HIV care and treatment [31, 43].

The study data were abstracted from HIV care facility clinical records. The absence of standard processes, guidelines, and diagnostic tools for screening and testing for NCDs at HIV care facilities resulted in our survey underestimating NCD burden. Data for all NCD categories, except for elevated blood pressure, were identified through documentation of diagnoses in clinic records. Notably, the majority of patients who were classified as having an NCD in this survey were identified through review of serial blood pressure measurements, and not through a documented history of hypertension in the medical record. Additionally, patients with conditions associated with high mortality such as stroke, myocardial infarction, and severe heart failure may be less likely to be identified through clinic records, either not reaching initial care or being lost-follow-up prior to diagnosis. The high proportion of patients lost to follow up in this cohort likely also resulted in an underestimated of NCD burden. The retrospective design of our study limited our analysis of risk factors for NCDs among PLHIV that would have bolstered our study findings and allowed us to make robust comparisons to other nationwide NCD surveys [44].

## Conclusions

We identified a high prevalence of NCDs among PLHIVs in Kenya that likely represents a substantial underdiagnoses of these categories of NCDs. Systematic screening and treatment for NCDs using standard guidelines should be integrated into HIV care and treatment programs in sub-Saharan Africa [2, 27]. Knowledge of NCDs burden could be improved through surveillance mechanisms and registries [45]. As Kenya seeks to reach the ambitious UNAIDS 90–90-90 goals through expanded treatment, strategies need to be developed that ensure health gains for PLHIVs are not eroded by a rising burden of NCD morbidity and mortality.

## Abbreviations

ABC: Abacavir
ART: Antiretroviral therapy
AZT: Zidovudine
CDC: Centers for Disease Control and Prevention
d4T: Stavudine
LPV/r: Lopinavir/Ritanovir
LSTIK: Longitudinal Surveillance of Treatment in Kenya
NASCOP: National AIDS and STI Control Program
NCD: Noncommunicable disease
PLHIV: People living with HIV
SSA: Sub-Saharan Africa
TDF: Tenofovir
UCSF: University of California, San Francisco
WHO: World Health Organization
